# Initiation of Resuscitation with High Tidal Volumes Causes Cerebral Hemodynamic Disturbance, Brain Inflammation and Injury in Preterm Lambs

**DOI:** 10.1371/journal.pone.0039535

**Published:** 2012-06-22

**Authors:** Graeme R. Polglase, Suzanne L. Miller, Samantha K. Barton, Ana A. Baburamani, Flora Y. Wong, James D. S. Aridas, Andrew W. Gill, Timothy J. M. Moss, Mary Tolcos, Martin Kluckow, Stuart B. Hooper

**Affiliations:** 1 The Ritchie Centre, Monash Institute of Medical Research, Monash University, Clayton, Victoria, Australia; 2 Department of Obstetrics and Gynecology, Monash University, Clayton, Victoria, Australia; 3 Centre for Neonatal Research and Education, School of Women’s and Infants’ Health, The University of Western Australia, Crawley, Western Australia, Australia; 4 Department of Neonatal Medicine, Royal North Shore Hospital and University of Sydney, Sydney, New South Wales, Australia; Erasmus University Rotterdam, The Netherlands

## Abstract

**Aims:**

Preterm infants can be inadvertently exposed to high tidal volumes (V_T_) in the delivery room, causing lung inflammation and injury, but little is known about their effects on the brain. The aim of this study was to compare an initial 15 min of high V_T_ resuscitation strategy to a less injurious resuscitation strategy on cerebral haemodynamics, inflammation and injury.

**Methods:**

Preterm lambs at 126 d gestation were surgically instrumented prior to receiving resuscitation with either: 1) High V_T_ targeting 10–12 mL/kg for the first 15 min (n = 6) or 2) a protective resuscitation strategy (Prot V_T_), consisting of prophylactic surfactant, a 20 s sustained inflation and a lower initial V_T_ (7 mL/kg; n = 6). Both groups were subsequently ventilated with a V_T_ 7 mL/kg. Blood gases, arterial pressures and carotid blood flows were recorded. Cerebral blood volume and oxygenation were assessed using near infrared spectroscopy. The brain was collected for biochemical and histologic assessment of inflammation, injury, vascular extravasation, hemorrhage and oxidative injury. Unventilated controls (UVC; n = 6) were used for comparison.

**Results:**

High V_T_ lambs had worse oxygenation and required greater ventilatory support than Prot V_T_ lambs. High V_T_ resulted in cerebral haemodynamic instability during the initial 15 min, adverse cerebral tissue oxygenation index and cerebral vasoparalysis. While both resuscitation strategies increased lung and brain inflammation and oxidative stress, High V_T_ resuscitation significantly amplified the effect (p = 0.014 and p<0.001). Vascular extravasation was evident in the brains of 60% of High V_T_ lambs, but not in UVC or Prot V_T_ lambs.

**Conclusion:**

High V_T_ resulted in greater cerebral haemodynamic instability, increased brain inflammation, oxidative stress and vascular extravasation than a Prot V_T_ strategy. The initiation of resuscitation targeting Prot V_T_ may reduce the severity of brain injury in preterm neonates.

## Introduction

Brain injury, particularly white matter injury and intracranial hemorrhage, is a major problem in very premature infants and the incidence and severity increases with decreasing gestational age and birth weight [1]. In addition to immaturity of the brain at the time of preterm birth, these babies may be exposed to systemic pathogenic factors such as infection/inflammation (both ante- and post-natal) and disturbed cerebral blood flow [2], [3], [4]. In the immediate newborn period, resuscitation, mechanical ventilation, poor ventricular function, rapid volume expansion and a patent ductus arteriosus are strongly associated with cerebral pathology in immature newborns [2], suggesting that early respiratory and cardiovascular care of preterm infants may critically influence the immature brain and the progression towards brain injury.

Recent clinical trials find that many preterm infants are inadvertently exposed to high tidal volumes (V_T_) during the initial resuscitation in the delivery room [5], [6]. The initiation of resuscitation is usually provided manually and the delivered V_T_ is poorly controlled and not routinely measured [7]. However, the initiation of respiratory support after birth, particularly with high V_T_, can induce an inflammatory response leading to chronic diseases of the lung such as bronchopulmonary dysplasia [8], [9].

We, and others, have shown that resuscitation with high V_T_ for 15 min initiates a pulmonary inflammatory response [10], [11], [12], which increases systemic proinflammatory cytokine mRNA expression [13], [14], [15]. Further, the initiation of ventilation with high airway pressures destabilizes the cardiopulmonary transition by increasing pulmonary vascular resistance, decreasing pulmonary blood flow, re-establishing right-to-left shunting through the ductus arteriosus and ultimately reducing left ventricular output [16]. These cardiovascular disturbances may underlie the pathogenesis of brain injury [17], acting via a reduction in cerebral blood flow indicated by reduced superior vena cava flow. Low SVC flow in the first 24 h after birth in preterm infants (<28 weeks), is strongly associated with IVH and long-term neurodevelopmental disability [18], [19]. We have recently shown that cerebral haemodynamics were most affected by resuscitation within the first 15 min [20], coinciding with the major events of the cardiopulmonary transition at birth [21].

Given that the initial resuscitation of the preterm neonate, particularly with the use of high V_T_, can result in a pulmonary and systemic inflammatory response and cause cardiopulmonary haemodynamic instability and hypoxia, we aimed to investigate the effects of resuscitation on the preterm brain. Specifically, we compared cerebral haemodynamic and brain histology using two different resuscitation strategies, one using high V_T_ and therefore considered injurious, versus a protective package of care, consisting of prophylactic surfactant, a sustained inflation followed by low V_T_ resuscitation, designed to optimize transition with minimal barotrauma.

## Materials and Methods

### Ethics Statement

The experimental protocol was performed in accordance with guidelines established by the National Health and Medical Research Council of Australia and was approved by the Monash Medical Centre (MMCA) animal ethics committee at Monash University.

### Instrumentation and Delivery

At 126±2 d (mean ± SD), the ewe and fetus were anesthetized (2% Isoflurane in oxygen, Bomac Animal Health, NSW, Australia) and the fetal head and neck were exposed *via* cesarean section. A skin incision was made in the fetal neck for placement of ultrasonic flow transducers (3 mm; Transonic Systems, Ithaca, NY) around the left and right carotid arteries. Carotid blood flow correlates highly with cerebral blood flow [22]. A polyvinyl catheter was placed into the left jugular vein. The fetal trachea was intubated with a cuffed endotracheal tube (4.0 mm) and lung liquid was drained passively. A transcutaneous oximeter (Masimo, Irvine, CA) was attached around the right forelimb before the umbilical cord was clamped and cut. Lambs were delivered, dried, weighed, placed under a radiant heater and resuscitation initiated (see below). Polyvinyl catheters were placed into an umbilical artery to measure mean arterial pressure. All lambs received sedation (Alfaxane i.v. 5–15 mg/kg/h; Jurox, East Tamaki, Auckland, New Zealand) in 5% dextrose to minimize spontaneous breathing during the experiment via the umbilical vein catheter. Left and right carotid arterial blood flows (CABF) were recorded continuously (Powerlab; ADInstruments, Castle Hill, NSW, Australia), along with mean arterial pressure (DTX Plus Transducer; Becton Dickinson, Singapore), from before delivery until the end of the experiment. Unventilated controls (UVC; n = 6) at the same age were delivered via caesarean section and immediately killed (sodium pentobarbitone: >100 mg/kg i.v.) without undergoing surgery or postnatal anaesthesia. Ewes were similarly humanely killed after delivery of the lambs.

### Resuscitation Strategy

Lambs were randomly assigned to receive either of two strategies that, based on previous studies, would be expected to either cause lung injury (High V_T_ group; n = 6) or to protect the lung from injury (Prot V_T_ group; n = 6). These were: 1) High V_T_, targeting a volume of 12–15 mL/kg within the first postnatal breaths using a neonatal positive pressure ventilator (Babylog 8000+, Dräger, Lübeck, Germany), which was maintained for 15 minutes to mirror the time taken for clinical neonatal resuscitation and transfer to NICU [23]; or 2) Prot V_T_, consisting of prophylactic surfactant (100 mg/kg, Curosurf^R^, Chiesi Pharma, Italy) followed by a 20 second sustained inflation delivered by a Neopuff (Fisher & Paykel Healthcare, Panmure, Auckland, New Zealand) using a peak inspiratory pressure (PIP) of 35 cmH_2_O. This second package of care was chosen to minimize pulmonary and systemic inflammation and improve the cardiopulmonary circulatory transition at birth [24], [25], [26], [27]. After the sustained inflation in the Prot V_T_ group and after 15 min of ventilation in High V_T_ group, all lambs were ventilated in volume guarantee mode (V_T_ 7 mL/kg; PEEP, 5 cmH_2_O; inspiratory time, 0.3 s; and expiratory time, 0.6 s) for the remainder of the experiment. The high V_T_ group did not receive surfactant. The total ventilation time for both groups was 90 minutes. In all lambs, ventilation was conducted using warmed, humidified air with the fraction of inspired oxygen initially set at 0.4 but adjusted to maintain arterial oxygen saturation (SaO_2_) between 88–95%. Well-being of the newborn lamb was monitored by regular blood gas analysis (ABL30, Radiometer, Copenhagen, Denmark) and pre-ductal transcutaneous oxyhemoglobin saturation.

Left ventricular output and the proportion of left-to-right to right-to-left shunting through the ductus arteriosus was detected using pulsed Doppler ultrasound as described previously [16]. The ductal shunt was calculated as a DA ratio (time left to right/total shunt time). Spatially resolved spectroscopy (SRS, NIRO 200 Spectrophotometer; Hamamatsu Photonics K.K., Hamamatsu City, Japan) was employed for continuous recording of cerebral oxygenation expressed as a tissue oxygenation index (TOI, %) at 6 Hz. The NIRO 200 also provides continuous measurements of changes in concentrations (µMolar.cm) of oxyhemoglobin (ΔHbO), deoxyhemoglobin (ΔHHb) and total haemoglobin (ΔHbT = ΔHbO+ΔHHb). Two aligned photodetectors are housed in the detection probe which is spaced 4 cm from the emission probe, with both the emission and detection probes placed over the fronto-parietal region and covered with light-proof dressing, as described previously [28]. Changes in Cerebral Blood Volume (ΔCBV, ml/100 g) were calculated from measurements of the ΔHbT, using a differential path-length factor of 4.99 [28], [29] and formula as shown below [30].

### Calculations

Dynamic compliance (C_dyn_), adjusted for weight, was calculated as *V*_T/_kg/(PIP - PEEP). Ventilatory efficiency index (VEI) was determined as 3800/(Δ*P* ·*f* ·*P*aCO_2_) where 3800 is a constant for the production of carbon dioxide (mL·mmHg·kg^−1^·min^−1^), Δ*P* = PIP-PEEP and *f* is the respiratory frequency [31]. Arterial oxygenation was described using the alveolar-arterial difference in oxygen (AaDO_2_) [32]. Oxygenation Index was calculated as OI  =  (F_i_O_2_ · mean airway pressure)/*P*aO_2_ · ΔCBV = (ΔHBT · MW_hemoglobin_·10^−6^)/(tHb · 10^−2^ · CLVHR · Dt · 10) where MWhemoglobin  =  molecular weight of hemoglobin  = 64,500, tHb  =  concentration of hemoglobin in large vessels in g · 100 mL^−1^, CLVHR  =  cerebral to large vessel hematocrit ratio  = 0.69, and Dt  =  brain tissue density in g·mL^−1^ = 1.05.

### Lung and Brain Collection and Processing

At the conclusion of the ventilatory protocol, lambs were humanely killed, weighed and the lungs and brains collected. All histological and molecular assessments of resuscitated lambs were compared to UVC to determine the comparative effects of different resuscitation strategies on lung and brain inflammation and injury.

The lung was removed and sections of the right lower lobe were snap-frozen in liquid nitrogen for subsequent measurement of interleukin (IL)-1β, IL-6 and IL-8 mRNA expression using quantitative RT-PCR [33].

The brain was removed and sectioned as described previously [34]. The left hemisphere was sectioned into approximately 4-mm slices, and fixed in 4% paraformaldehyde for histological and immunohistochemical analyses. Serial sections (10 µm) at the level of the lateral ventricle were stained with: hemotoxylin and eosin (H&E) for gross assessment of brain pathology. Immunohistochemistry was used to assess: the presence of inflammatory cells using peroxidase-labeled lectin (1∶200, Sigma, USA); lipid peroxidation (4-Hydroxynonenal; 4HNE, 1∶1000, Calbiochem, USA); and blood brain barrier integrity (serum albumin, 1∶1000, Accurate Chemical & Scientific Corporation, USA). Lectin- and 4HNE-positive cells were counted in four random non-overlapping high-powered fields in subcortical and periventricular white matter by a blinded assessor and expressed as number of positive cells/mm^2^ (ImageJ; NIH image, Bethesda, Maryland, USA). H&E and albumin analysis was conducted using six random high-powered fields per section, 2 sections per lamb, from the deep periventricular or subcortical white matter (DP2-BSW, Olympus, Japan). For H&E sections, each field was designated a qualitative score for the assessment of brain injury, where 0 =  none, 1 =  mild (<50% of the field of view occupied by brain damage), 2 =  severe (>50% of the field of view occupied by brain damage). Albumin extravasation was determined by the presence or absence of albumin within the tissue surrounding blood vessels; only the incidence was noted.

### Statistical Analysis

Data are presented as mean ± SEM. Serial data were compared between groups using two-way ANOVA with repeated measures (Sigmastat v3.0, SPSS Inc.). The first 15 minutes was analysed separated as above. Posthoc comparisons were performed using the Holm-Sidak method. ANOVA was used to compare biochemical and histologic indices of injury. Statistical significance was accepted for p<0.05.

## Results

Umbilical arterial blood gas and acid/base status at birth were normal for all lambs and not different between groups (Mean for all groups: pH: 7.19±0.05 PaCO_2_: 67.0±5.8 mmHg: PaO_2_: 28.8±5.1 mmHg; SaO_2_: 60.3±8.7%). Lamb body weights were not different between groups (UVC: 2.9±0.2 kg; Prot V_T_: 3.2±0.2 kg; High V_T_: 3.1±0.2 kg; p = 0.608).

### Ventilation and Oxygenation

**Figure 1:**
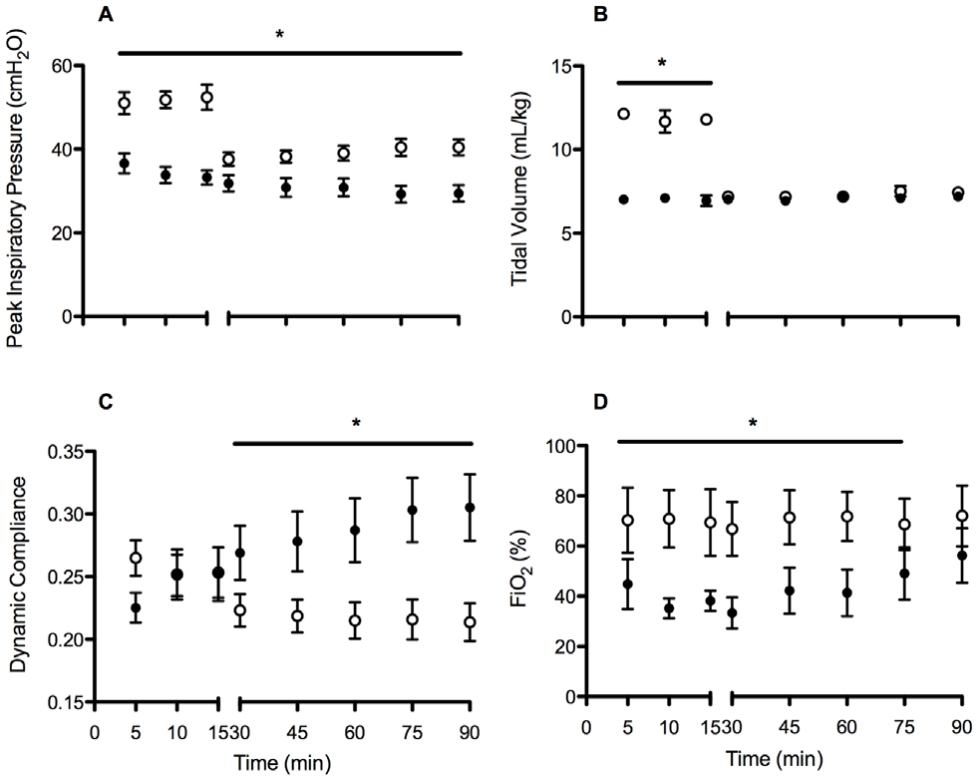
Peak inspiratory pressure (A), tidal volume (B), dynamic compliance (C) and the fraction of inspired oxygen (FiO2; D) in High V_T_ (open circles) and Prot V_T_ (closed circles) lambs. *p<0.05 High V_T_ vs. Prot V_T_.

Peak inspiratory pressure was higher in the High V_T_ group during the initial 15 min resuscitation period, as expected, and remained higher for the subsequent 75 min ventilation period (p = 0.002; Figure 1A) compared to the Prot V_T_ group. The mean tidal volume obtained in the High V_T_ group in the first 15 minutes was 12.5±0.3 mL/kg (Range 10.9–13.4 mL/kg) while in the Prot V_T_ group it was 7.0±0.2 (Range 6.7–7.2 mL/kg; p<0.001; Figure 1B). V_T_ was not different after the initial 15 minutes. Lung static compliance was lower (worse) in High V_T_ lambs from 30 min (p<0.001; Figure 1C), while required FiO_2_ was higher in High V_T_ lambs throughout the ventilation procedure (p = 0.028; Figure 1D). Calculated airway resistance was significantly higher in High V_T_ lambs compared to Prot V_T_ group lambs (High V_T_: 82.1±4.9, Prot V_T_: 48.7±5.4; p = 0.002).

**Figure 2:**
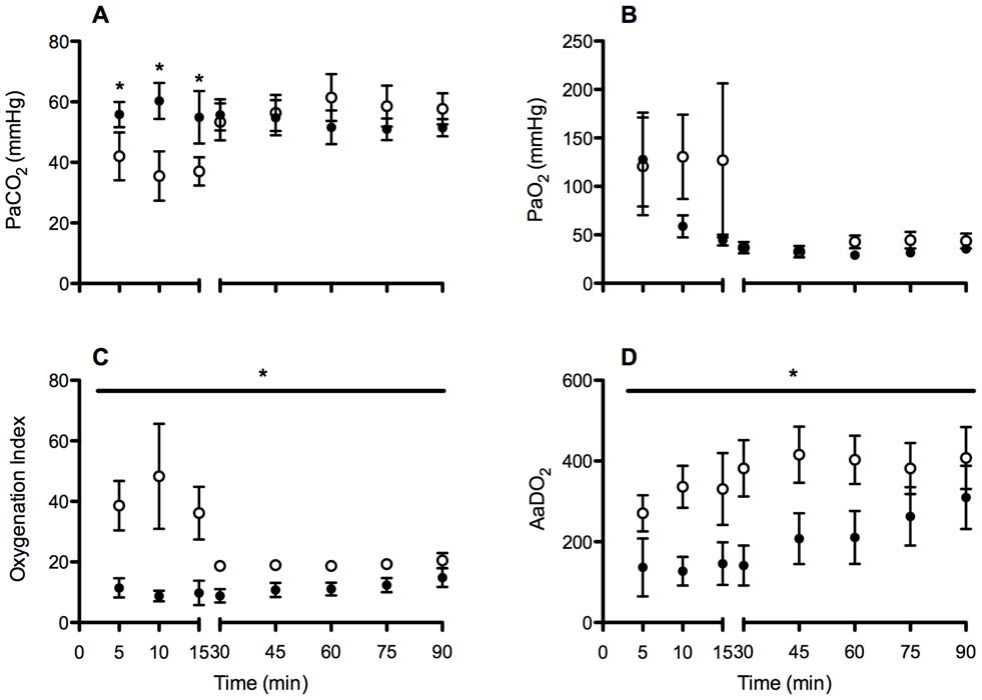
The partial pressure of arterial (Pa) carbon dioxide (CO_2_; A), oxygen (O_2_; B), oxygenation index (C) and the alveolar-arterial difference in oxygen (AaDO_2_; D) in High V_T_ (open circles) and Prot V_T_ (closed circles) lambs. *p<0.05 High V_T_ vs. Prot V_T_.

The partial pressure of arterial oxygen (*P*aO_2_) was higher at 10 and 15 min in High V_T_ lambs but variability precluded significance (p = 0.266; Figure 2A). *P*aCO_2_ was significantly lower in High V_T_ lambs during the 15 min resuscitation period (p<0.001; Figure 2B), but was not different thereafter. Oxygenation Index was higher (p = 0.003; Figure 2C) and the alveolar-arterial difference in oxygen was lower (p = 0.039; Figure 2D) throughout resuscitation and ventilation in High V_T_ lambs, indicative of poorer oxygenation. Arterial oxygen saturation was successfully maintained in both groups (High V_T_: 87.1±2.8%, Prot V_T_: 88.1±2.9%) and was not different between groups (p = 0.393). Calculated cerebral oxygen delivery was not different between groups at any of the blood-gas time points (p = 0.840). Body temperature (p = 0.784), blood lactate (p = 0.893) or blood glucose (p = 0.435) were not different between groups (data not shown).

### Hemodynamics

**Figure 3:**
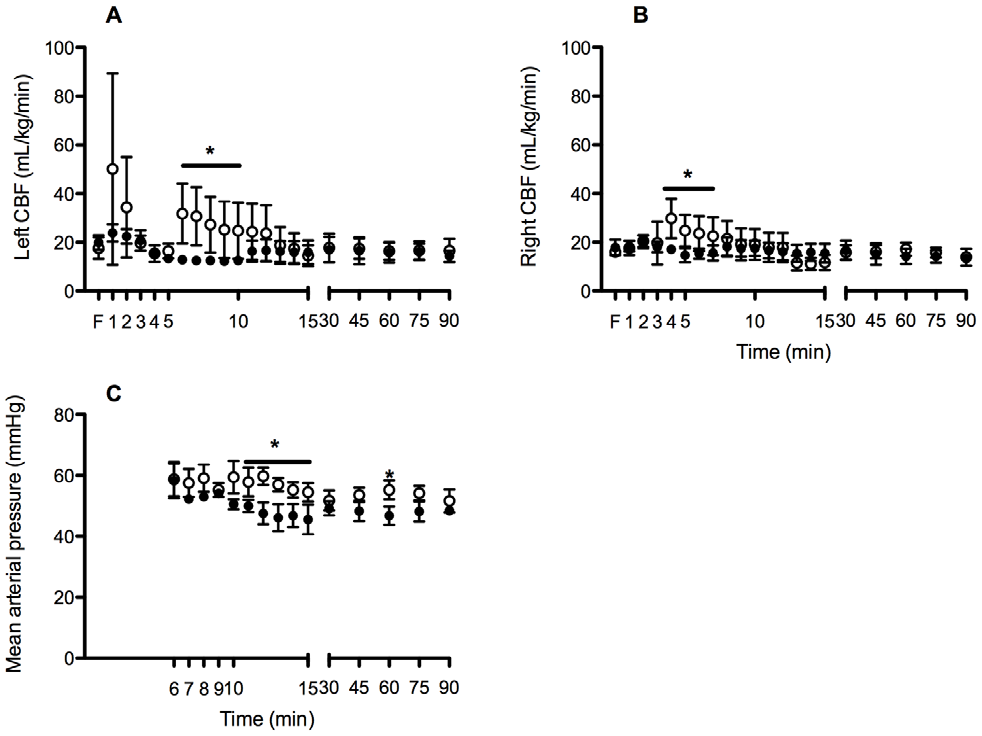
Left (A) and right (B) carotid blood flow (CABF) and mean arterial pressure (C) in High V_T_ (open circles) and Prot V_T_ (closed circles) lambs. *p<0.05 High V_T_ vs. Prot V_T_.

Combined left and right CaBF were significantly higher within the first 10 min in High V_T_ lambs compared with Prot V_T_ lambs (Fig 3 A & B). The coefficient of variation in total CaBF was significantly higher in High V_T_ lambs compared to Prot V_T_ (mean: High V_T_: 0.60±0.06 vs. Prot V_T_ 0.38±0.05; p<0.01); the majority of the variability was observed within the first 15 min. Maximum CaBF (p<0.001) and the amplitude of CaBF (p = 0.002) was significantly higher within the first 15 min in High V_T_ lambs compared to Prot V_T_. Mean arterial pressure was significantly higher in High V_T_ lambs between 10–15 min and at 60 min compared to Prot V_T_ lambs (Fig 3C). Left ventricular output (p = 0.57), heart rate (p = 0.14) and DA Ratio (p = 0.28) were not different between groups. LVO and heart rate decreased similarly in both groups over time (p<0.001).

### SRS-NIRS

**Figure 4:**
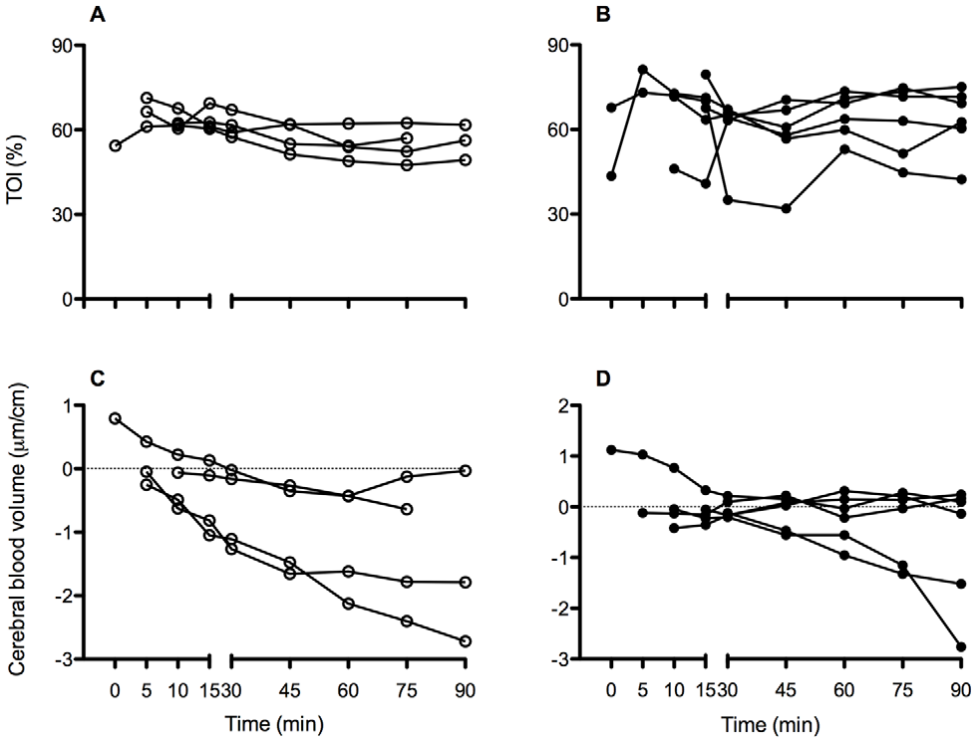
Tissue oxygenation index (TOI; A & B) and cerebral blood volume (C & D) in High V_T_ (open circles) and Prot V_T_ (closed circles) lambs. Values are derived using spatially resolved near infrared spectroscopy. Note the greater variability in TOI within the first 15 min during High V_T_ lambs (B) compared to Prot V_T_. Cerebral vascular vasoparalysis is evident in 4 of 5 High V_T_ lambs by the maintenance of near stable cerebral blood volume (D).

NIRS data was successfully obtained from 4 Prot V_T_ and 6 High V_T_ lambs. Averaged Tissue Oxygenation Index (Figure 4A & B) was not different between groups, but TOI variations between and within individual lambs were much greater in the High V_T_ group. Changes in cerebral blood volume from baseline measurements was significantly lower in High V_T_ lambs compared to Prot V_T_ lambs from 60 min (p<0.001; Figure 4C & D). The Prot V_T_ group showed a significant fall in CBV from baseline (p<0.001), while the high V_T_ group remained unchanged.

### Inflammation and Oxidative Injury

**Figure 5:**
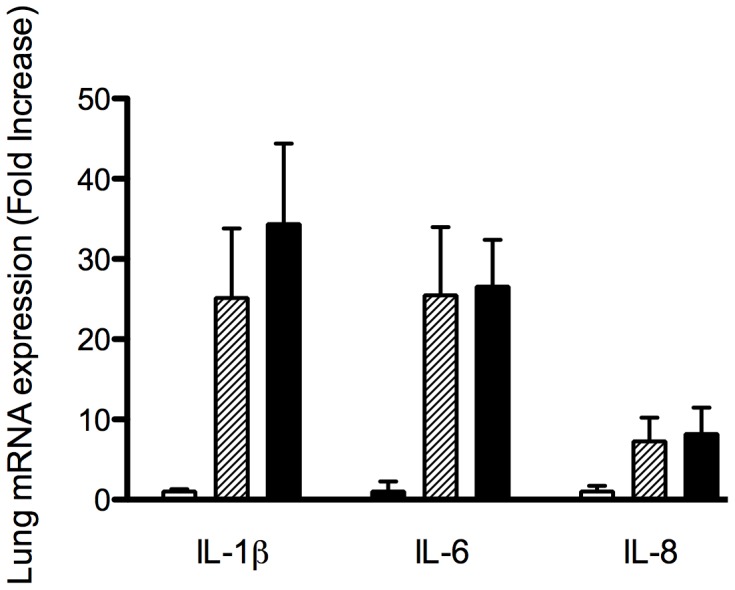
Lung pro-inflammatory mRNA cytokine expression of interleukin (IL)-1β, IL-6 and IL-8 in unventilated controls (open bars), Prot V_T_ (shaded bars) and High V_T_ (black bars) lambs. Values are expressed relative to UVC. Resuscitation, irrespective of the strategy, increased lung inflammation. *p<0.05 vs UVC.

Lung mRNA cytokine expression of IL-1β, IL-6 and IL-8 was significantly elevated in High V_T_ lambs compared to Prot V_T_ lambs and UVC (Fig 5). Within the subcortical white matter of the brain, the number of lectin-positive cells was significantly higher in the High V_T_ group compared to UVC (p = 0.014; Table 1). The Prot V_T_ group was not different to UVC (p = 0.072). There were no differences observed in the periventricular white matter between the groups (p = 0.271). The number of 4HNE-positive cells, indicative of oxidative injury, was significantly higher in High V_T_ lambs within the periventricular white matter compared to UVC (p<0.001) and showed a strong trend to be greater than Prot V_T_ lambs (p = 0.054). There was no difference observed in the subcortical white matter (p = 0.13; Table 1).

**Table 1:** Assessment of brain injury.

Group	UVC (n = 6)			Prot V_T_ (n = 5)			High V_T_ (n = 5)		
Incidence and severity of injury (%)	Incidence	Mild	Severe	Incidence	Mild	Severe	Incidence	Mild	Severe
**Gross Anatomical Injury**									
Periventricular white matter	0	0	0	40	20	20	20	20	0
Subcortical white matter	12	0	12	40	40	40	40	20	20
**Lipid Peroxidation**									
Periventricular white matter (cells/mm^2^)	245±99			412±41			580±60*^#^		
Subcortical white matter (cells/mm^2^)	271±73			393±38			402±39		
**Vascular protein extravasation**									
Periventricular & subcortical white matter (%)	0			0			60*^#^		
**Infiltrating inflammatory cells**									
Periventricular white matter (cells/mm^2^)	120±37			205±26			211±65		
Subcortical white matter (cells/mm^2^)	196±58			536±40			728±242*		

Incidence and severity of gross anatomical brain injury expressed as % of the lambs displaying evidence of mild or severe injury. *indicates significant difference between High V_T_ and UVC. ^#^indicates significant difference between High V_T_ and Prot V_T_.

### Brain Pathology and Hemorrhage

The incidence and severity of gross anatomical brain injury within the subcortical and periventricular white matter was higher in resuscitation groups compared to UVC, but no difference was observed between resuscitation groups receiving High V_T_ compared to Prot V_T_ (Table 1). The incidence of vascular extravasation, as evidenced by protein leakage, was evident in 60% of High V_T_ lambs, but was not observed in Prot V_T_ or UVC (p = 0.030; Table 1).

## Discussion

Our previous studies suggest that the initial 15 minutes of resuscitation after birth may be a critical time period for the development of brain inflammation and injury in preterm infants [20]. The findings of our current study are consistent with this contention and further demonstrate that the ventilation strategy used in this critical time period after birth can adversely affect the developing brain. Importantly, the use of high tidal volumes for resuscitation alters cerebral haemodynamics, increases brain inflammation, oxidative stress and vascular extravasation compared to a Prot V_T_ protective resuscitation protocol.

Although preterm infants can be inadvertently exposed to high V_T_ during the initial resuscitation in the delivery room [5], [6] that can result in lung inflammation and injury [8], [9], [11], [14], the consequences for the brain have not been investigated. The principal pathogenic factors causing neonatal preterm brain injury are infection/inflammation (both ante- and post-natal) and disturbed cerebral blood flow [2], [3], [4]. These factors can occur from aggressive resuscitation of preterm neonates [14], [20], which can also adversely affect and destabilize cardiac function leading to a much greater risk of cerebral vascular injury.

In the Prot V_T_ group we aimed to achieve stable arterial blood gas levels and to avoid large fluctuations in intra-thoracic pressure that disturb cardiac function. We subsequently showed that cerebral oxygenation was also relatively stable and that CBV gradually reduced in all Prot V_T_ lambs. The normal postnatal reduction in cerebral blood volume and cerebral blood flow is a well-established and described phenomenon in both animals [35] and humans at term [36], [37] and preterm [38]. It results from the normal postnatal increase in oxygen content and demonstrates the importance of oxygen delivery in regulating CBF [39]. In contrast, lambs subjected to High V_T_ resuscitation failed to display a decrease in CBV, despite similar or higher (first 15 min) blood oxygenation levels, suggesting that the normal postnatal adaptation of the cerebral circulation has been altered in these lambs. Furthermore, as these lambs also had lower *P*aCO_2_ levels during the 1^st^ 15 min, we would expect this to enhance the cerebrovasoconstriction caused by increased oxygenation, providing further evidence that the cerebral vascular response was abnormal in High V_T_ lambs. It is possible that this reflects injury to the cerebral vessels and likely contributed to the vascular leakage observed in the High V_T_ group, particularly as relative cerebral hyperemia and cerebral vasoparalysis are known features in neonatal brain injury [40], [41]. Our data on the carotid blood flow variations also support the findings of IVH in 21 of 23 infants in which fluctuations in cerebral blood flow velocity (measured by Doppler ultrasound) were detected [42]. Coupled with our previous study [20], our findings show that cerebral haemodynamics during the immediate newborn period is critically dependent upon pulmonary blood flow and LVO, which in turn, are critically dependent on resuscitation strategy.

Our study highlights the ability to detect some aspects of brain injury, including inflammation, oxidative stress and microscopic hemorrhage in brains within 90 minutes after the onset of resuscitation/ventilation at birth. This has not been previously reported, with most commentaries indicating that it takes 24 to 48 hours before brain pathologies can be detected. In the clinical setting, most cerebral ultrasounds are performed more than 24 hours after birth, with MRI studies conducted much later, and only if the baby can be moved. The study protocol allowed us to determine the effects of the initial resuscitation strategy, without the confounding influences of prolonged maintenance of respiratory and life support and other postnatal influences including nutrition and sepsis.

To assess how High V_T_ resuscitation impacts the immature brain, we compared these lambs with lambs resuscitated with a strategy we considered to be less injurious (Prot V_T_) and to minimally interfere with the cardiovascular transition at birth. The Prot V_T_ group received prophylactic surfactant, a 20 second sustained inflation followed by volume guarantee ventilation at 7 mL/kg, representing the normal spontaneous tidal volume of preterm lambs [15]. We have previously shown that an initial sustained inflation (20 s) can fully aerate the lung before the onset of tidal breathing [24], thereby facilitating a smooth pulmonary blood flow transition immediately after birth [24], decreases early markers of lung injury and inflammation [25] and improves CaBF stability compared to controls [26]. Further, prophylactic surfactant administration decreases markers of lung inflammation caused by high tidal volumes at birth [27] and markedly improves the uniformity of lung aeration [43]. While resuscitation in both groups increased indices of brain injury, inflammation and oxidative stress, the Prot V_T_ group displayed normal postnatal adaptive changes within the cerebral circulation and had reduced brain inflammation compared to the more injurious group. This suggests that improved resuscitation procedures, including appropriate lung recruitment and minimizing lung injury, may reduce the risk and severity of brain injury.

This study confirms that resuscitation with high V_T_ for 15 min initiates a pulmonary inflammatory response [10], [12], [44] which increases systemic proinflammatory cytokine mRNA expression [13], [14], [15]. One of the major up-stream mechanisms of brain injury, particularly pertaining to cerebral white matter injury of prematurity, is inflammation/infection, mediated by a systemic up-regulation of inflammatory cytokines [3]. While most of the research on the role of pro-inflammatory cytokines in white matter injury has focused on intrauterine inflammation or postnatal sepsis, our previous work suggests there may be a contribution from resuscitation [20]. In this study, 15 min of high V_T_ resuscitation increased biomarkers of inflammation within the lung and brain compared to the Prot V_T_ group. Up-regulation of systemic pro-inflammatory cytokines (IL-1β, IL-6 and TNF-α) are known to compromise integrity of the blood brain barrier and neuro-vascular circulation [45]. We observed protein extravasation in 60% of high V_T_ lambs, which was not present in unventilated control or Prot V_T_ lambs, confirming a strong association between increased systemic cytokines levels and blood brain barrier integrity.

Oxidative stress is another notable mechanism implicated in white matter injury, such as periventricular leukomalacia (PVL) [3]. The neuroglial oligodendrocyte cells function to myelinate developing axons and their precursors are exquisitely sensitive to oxidative injury [46]. Thus, oxidative injury impairs myelination, which is an important pathological feature of PVL. Clinical studies have found increased lipid peroxidation in cases of PVL [47]. Our study showed that high V_T_ lambs had elevated cellular lipid peroxidation within the periventricular white matter, potentially resulting in increased white matter injury within this group. The findings of increased brain inflammation and oxidative stress resulting from resuscitation, and particularly high V_T_ resuscitation, indicates that the immediate resuscitation period in the delivery room is likely a critical window for the development of white matter injury.

In summary, this study has shown that resuscitation increases brain inflammation and oxidative stress, but an initial high V_T_ resuscitation strategy further exacerbated cerebral hemodynamic instability, brain inflammation and injury. This study highlights the critical role that the initial respiratory support has on the development of brain inflammation and injury, and the requirement for better monitoring of delivered tidal volumes to preterm infants in the delivery room. It also demonstrates that brain injury can be seen within 90 minutes of the onset of an injurious stimulus, and the severity of brain injury can be reduced by protective resuscitation strategies in the delivery room.
